# The Dusseldorf Illustrated Schema Questionnaire for Children (DISC)

**DOI:** 10.1186/s41155-018-0087-y

**Published:** 2018-02-20

**Authors:** Christof Loose, Frank Meyer, Reinhard Pietrowsky

**Affiliations:** Department of Clinical Psychology, Institute of Experimental Psychology, Heinrich-Heine-University Dusseldorf, Universitatsstr. 1, 40225 Dusseldorf, Germany

**Keywords:** Early maladaptive schemas, Children, Questionnaire, Cartoons

## Abstract

Childhood experiences are considered to be of crucial importance in the formation of maladaptive schemas, according to Young’s concept. Although some schema questionnaires already exist for children, these instruments differ in their schema structures with between 8 and 12 identified factors. To obtain a deeper understanding of early maladaptive schemas in childhood an instrument based on Young’s 18-schema model was constructed (Dusseldorf Illustrated Schema Questionnaire for Children (DISC)).

Cartoons were designed which represented each schema, providing children with a visual impression of what was meant by the schema, and thus the questions that they had to answer. The items were phrased as self-report statements and children’s approval of the statements was assessed using a 4-point rating scale. The resulting preliminary questionnaire (18 cartoons, 90 items) was presented in classrooms across different school types (*N* = 569, between 8 and 13 years). A confirmatory factor analysis (CFA) was performed on this data to confirm the factorial structure of the questionnaire and to reduce the number of items to two items per schema. The DISC in its final version included 36 items and showed a sufficiently high test-retest reliability and convergent validity when assessed in comparison to another schema questionnaire for children. In addition, the present test is of predictive value since the DISC sum score correlated with ratings on the children’s behavioral problems. CFA showed a satisfactory goodness-of-fit based on the original 18-factor model, providing a compact instrument to assess schema representations and to evaluate the dynamics of maladaptation during child development.

## Background

Schema therapy is a psychotherapy that combines different therapeutic approaches: cognitive-behavioral therapy, psychodynamic approaches, Gestalt therapy, hypnotherapy, and humanistic therapies (Young, Klosko, & Weishaar, 2003). It has recently gained increased attention, since outcome studies have demonstrated its efficacy and effectiveness, especially in patients with personality disorders (Jacob & Arntz, 2013).

Beck (1967) defined a schema as a “structure for screening, coding and evaluating stimuli that impinge on the organism”. Young extended the term to include early maladaptive schemas (EMS) which can be regarded as distilled knowledge from unmet or frustrated emotional needs (synonym: core needs, psychological needs) early in life. This knowledge, which is stored in EMS, is defined as “pervasive and stable themes regarding oneself and one’s relationship with others” (Young et al., 2003, p. 7). Young proposed that ongoing noxious experiences with caregivers and significant others during childhood and adolescence, in interaction with temperamental factors, could not only result in the formation of EMS but also lead to dysfunctional behavior. Consequently, when specific EMS are triggered by a certain situation, which exhibits any similarities to aspects of the previous noxious experiences, the individual may react to this trigger with maladaptive or dysfunctional coping behavior, i.e., surrender, avoidance, or overcompensation. This dysfunctional coping behavior perpetuates the EMS, which leads to a greater risk of psychopathology (McGinn & Young, 1996; Young et al., 2003).

For adults, there has been ample research, which has shown that the prevalence of EMS has a substantial correlation with mental disorders, behavioral problems, and personality disorders (Jovev & Jackson, 2004; Nordahl, Holthe, & Haugum, 2005). Similarly, for adolescents and children, there have been a substantial amount of studies which have indicated a strong link between EMS and psychopathological symptoms and mental disorders (Bakshi Bojed & Nikmanesh, 2013; Calvete, 2008, 2014; Calvete, Orue, & Hankin, 2013a, 2013b, 2015; Damiano, Reece, Reid, Atkins, & Patton, 2015; González-Jiménes & del Mar Hernández-Romera, 2014; Lawrence, Allen, & Chanen, 2011; Lumley & Harkness, 2007; Muris, 2006; Orue, Calvete, & Padilla, 2014; Richardson, 2005; Roelofs, Lee, Ruijten, & Lobbestael, 2011; Roelofs, Onckels, & Muris, 2013; Simard, Moss, & Pascuzzo, 2011; Van Vlierberghe & Braet, 2007; Van Vlierberghe, Braet, Bosmans, Rosseel, & Bögels, 2010).

Young and Brown (1990) first proposed 16 EMS, based on clinical experiences with chronic psychotherapy patients. Schmidt, Joiner, Young, and Telch (1995) followed by confirming a 15-factor structure; this list of schemas was then expanded to 18 EMS (Young, 1998). Young grouped the 18 EMS into five schema domains: disconnection and rejection, impaired autonomy and performance, impaired limits, other-directedness, and over-vigilance and inhibition (Young et al., 2003). These domains are associated with basic emotional needs such as attachment, autonomy, self-esteem, pleasure, and structure/limit setting (Young et al., 2003). The frustration or inadequate fulfillment of these needs will lead—along with the background of temperamental as well as model and operant learning factors—to the development of EMS. In Table 1, the correspondence of EMS to schema domains and to emotional needs is depicted (for a more detailed description of EMS, domains, and needs, see Arntz & Jacob, 2012; Rafaeli, Bernstein, & Young, 2011; Young et al., 2003).

**Table 1:** Early maladaptive schemas and their correspondence to schema domains and unmet needs

Schemas		Domains	Unmet needs
1	Abandonment/instability (AB)	Disconnection and rejection	Attachment
2	Mistrust/abuse (MA)		
3	Emotional deprivation (ED)		
4	Defectiveness/shame (DS)		
5	Social isolation/alienation (SI)		
6	Dependence/incompetence (DI)	Impaired autonomy and performance	Autonomy/self-efficacy
7	Vulnerability (VU)		
8	Enmeshment/undeveloped self (EU)		
9	Failure (FA)		
10	Entitlement/grandiosity (ET)	Impaired limits	Identity/structure/limits
11	Insufficient self-control/self-discipline (IS)		
12	Subjugation (SU)	Other-directedness	Self-esteem acceptance Autonomy/self-determination
13	Self-sacrifice (SS)		
14	Approval-seeking/recognition-seeking (AS)		
15	Negativity/pessimism (NP)	Over-vigilance and inhibition	Pleasure, spontaneity and play/fun
16	Emotional inhibition (EI)		
17	Unrelenting standards (US)		
18	Punitiveness (PU)		

Concerning diagnostic inventories, EMS have been studied with different versions of the Young Schema Questionnaire (YSQ; Arntz & Jacob, 2012; Rafaeli et al., 2011; Young, 1994, 2005a, 2005b; Young et al., 2003). Although the factorial structure of the YSQ may differ slightly from study to study, between 12 and 15 EMS have been identified by factor analysis for the questionnaires that included 15 to 16 EMS (Lee, Taylor, & Dunn, 1999; Rijkeboer & van den Bergh, 2006; Stopa, Thorne, Waters, & Preston, 2001; Waller, Meyer, & Ohanian, 2001; Welburn, Coristine, Dagg, Pontefract, & Jordan, 2002). Nevertheless, when using the third version of the YSQ composed of 18 EMS (YSQ-S3R; Young, 2005a, 2005b), all of these 18 EMS were verified via factor analysis (Calvete, Orue, & Gonzalez-Diez, 2013; Hawke & Provencher, 2012; Kriston, Schäfer, Jacob, Härter, & Hölzel, 2013; Lee, Choi, Rim, Won, & Lee, 2015; Saariaho, Saariaho, Karila, & Joukamaa, 2009). In adolescents, EMSs have also been investigated with a shorter form of the YSQ (YSQ-sf; Young, 1998) or an adapted version of the YSQ for adolescents (YSQ-A; Van Vlierberghe et al., 2010). Both questionnaires followed a 15-schema structure. The results of factorial analysis for this questionnaire were comparable with the studies on adults (who followed the 15/16-schema structure notion), suggesting a maximum of 15 factors in this population (adolescents). Another study into EMS prevalence in adolescents was carried out by Beckley (2002), who administered the YSQ-sf (Young, 1998) to a non-clinical sample of 705 teenagers aged 11 to 16. She also found a 15-factor structure solution within this younger population, comparable to that obtained with adolescents.

Also, in children, some studies have investigated EMS. According to Young’s assumption that EMS are distilled knowledge from unmet or frustrated emotional needs early in life, it is not clear *when* in childhood the formation of EMS may take place and whether all schemas, which exist at this early stage of life, are in their nature maladaptive.

The first published study about EMS in childhood with a child version of the YSQ—at least to our knowledge—was conducted by Stallard and Rayner (2005), who developed the Schema Questionnaire for Children (SQC; *n* = 47, ages 11–16). They also followed the 15-schema structure notion and created one item for each schema. The children filled out two questionnaires, the SQC and the YSQ-sf. The results were that 10 out of the 15 schemas in the SQC correlated significantly with the result of the YSQ-sf (Young, 1998); besides that, two more items containing almost significant coefficients were found. Stallard (2007) tested a 12-schema version of the SQC and was able to discriminate whether the participating child/teenager belonged to a non-clinical (*n* = 46, ages 11–16) or clinical sample (*n* = 53, ages 9–18). Within the same study, the test-retest reliability of the items was investigated by assessing EMS twice in a sample of 77 schoolchildren (ages 9–10) over a 6-month-interval. Correlation coefficients were moderate (range *r =* .27–.54), suggesting that the prevalence of specific schemas at one point is moderately stable over a period of at least 6 months.

Rijkeboer and de Boo (2010) also developed a schema questionnaire for children, the Schema Inventory for Children (SIC), that was also based on the early 15-schema model. They investigated the SIC’s psychometric properties in a non-clinical sample of children (*N* = 578, ages 8–13). Confirmatory factor analyses yielded satisfying fits for a modified model that included eight of the original 15 schemas, as well as three new factors, each containing a combination of two or three original schemas. Besides that, the authors found a strong relationship between most of their 11 SIC factors and a psychopathology questionnaire (adapted version of Early Adolescent Temperament Questionnaire-Revised, EATQ-R; Ellis & Rothbart, 2001). However, on two factors, an opposite pattern of associations emerged (i.e., enmeshment and self-sacrifice), suggesting that these schemas are not yet maladaptive, or at least would not have led to problematic scores on the EATQ-R. Another result of the study was, which the test-retest reliability (4 weeks’ time interval, *N* = 245) revealed Pearson’s correlations between *r* = .53 and *r* = .79 with an average of *r* = .67, indicating that the SIC represents reliable constructs.

Güner (2016) recently developed a new schema questionnaire (SQS; Early Maladaptive Schema Questionnaires Set for Children and Adolescents) and investigated 983 children (ages 10–16) with 97 items. She investigated all 18 original EMS using exploratory and confirmatory factor analyses and found 14 factors plus a new one (self-disapproval). The SQS showed good fit indices, high internal consistency, and consistency over a 1-month interval. Moreover, it significantly differentiated between children who had clinical diagnoses (*n* = 78) and children who had no diagnosis (*n* = 100). Concerning the issue of whether some schemas might be non-maladaptive, Güner reported for all investigated schemas significant correlations with subscales of the Symptom Assessment Questionnaire (SA-45, Strategic Advantage Inc. [SAI], 2000), underpinning the notion of their maladaptive nature.

To sum up, Young’s original 18 schemas were replicated with factor analyses for adults in various studies (Calvete, Orue, & Gonzalez-Diez, 2013; Hawke & Provencher, 2012; Kriston et al., 2013; Lee et al., 2015; Saariaho et al., 2009), and studies with adolescents have shown comparable results (Lumley & Harkness, 2007; Muris, 2006; Van Vlierberghe & Braet, 2007). In studies with children, many schemas (i.e., 12 from 15; Stallard & Rayner, 2005) and 11 (composed) of 15 schemas (Rijkeboer & de Boo, 2010) were also confirmed by factorial analyses. Beside the fact that other studies were also able to find and confirm largely all of the schemas they had investigated (Beckley, 2002; Schmidt et al., 1995), we intended to challenge this 18-factor notion for the investigation of EMS in childhood again, but unlike in Güner’s study, with the aid of pictorial representations of the schemas and in a more economical way by using two items per schema.

In addition, we intended to investigate the test-retest reliability of the questionnaire over two different time intervals (13–14 months and 24–36 months). Concurrent validity of the DISC will be investigated by comparing its results with those of the aforementioned SIC (Rijkeboer & de Boo, 2010). Finally, the predictive validity of the DISC will be assessed by correlating the DISC sum score with the total difficulties scores of the (self-reported and other-reported) Strength and Difficulties Questionnaire (SDQ; Goodman, 1997) which is also an indicator in the question of whether EMS are already maladaptive in children.

## Methods

### Participants

For the recruitment of participants, a total of 101 schools in Dusseldorf (Germany) and surrounding cities (radius, 150 km) were contacted, whereby 13 schools agreed upon participation (response rate, ~ 13%), including grammar schools, middle schools, secondary schools, and community and comprehensive schools, as well as primary (elementary) schools. All institutions were selected at random. With no specific exclusion criteria for participation, an opt-in recruitment process was applied, i.e., each child was able to participate in the study, given that one parent provided their written consent.

The investigated children represented a community sample (*N* = 569) of fourth graders (about 8- to 9-year-olds) to seventh graders (about 12- to 13-year-olds). With 51% (*n* = 290) being female, the gender ratio was therefore balanced. Forty-six children (8.1%) were fourth graders, 169 (29.7%) fifth graders, 197 sixth graders (34.6%), and 157 (27.6%) seventh graders. To ensure anonymity, the ethnic and national backgrounds as well as the exact ages of the children were not recorded.

Regarding different types of schools, 300 children (52.7%) attended a grammar school (Gymnasium), 76 (13.4%) a middle school (Realschule), and 21 (3.7%) a secondary modern school (Hauptschule). Furthermore, 88 children (15.5%) attended a comprehensive school (Gesamtschule) and 38 (6.7%) a community school (Gemeinschaftsschule), both of these school types representing a school community which integrates grammar, middle, and secondary modern schools into one school system. Finally, 46 (8.1%) attended the fourth grade of an elementary school (Grundschule). Schools with facilities for special needs education (e.g., for children with learning difficulties) were considered a special case and not included for reasons of homogenous sampling. The investigation period lasted from November 2012 to May 2016. The study was approved by the local ethics committee of the University of Dusseldorf.

### Measures

#### Dusseldorf Illustrated Schema Questionnaire for Children (DISC)

On the basis of the Young Schema Questionnaire (YSQ-S3R; Young, 2005b), the DISC was constructed to assess maladaptive schemas in children. For each of the 18 schemas proposed by Young, a pool of about ten items was created, oriented on the YSQ-items, but adapted for the children’s age. Five to seven of these ten items per schema were preselected by experts (experienced cognitive-behavioral therapists for children and adolescents, and/or schema therapists for children, with a minimum of 5 years of experience). The experts were asked to rate the conceptual fitness of each item on a scale ranging from 1 (“not fitting”) to 6 (“optimal fitting”). While assuring that all aspects of each schema were covered, the best-rated items per schema (in most cases rated 5 or 6) were chosen and then presented to three experienced primary school teachers (with a minimum of 5 years of experience) to assess whether the diction and phrasing was comprehensible to fourth graders. Finally, the best fitting five items in conceptual and comprehensive terms (chosen by the authors) were then evaluated by four fourth graders using the Cognitive Survey-technique (Prüfer & Rexroth, 2005), thus testing whether children of this age could comprehend the items in the intended way. Regarding the comprehensiveness, all items presented to the children were correctly understood and therefore approved for further investigation in the preliminary version of our questionnaire.

**Fig. 1:**
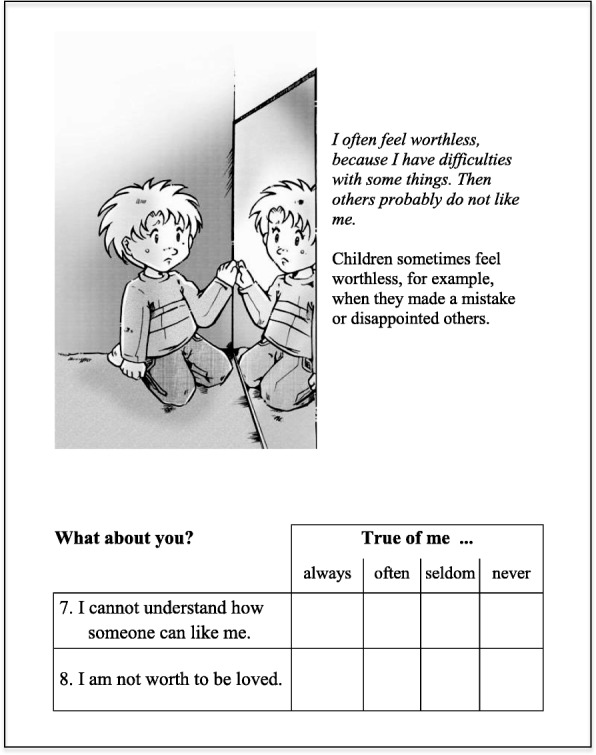
The schema defectiveness/shame with a cartoon, additional explanation of what is meant and the two items, selected for the DISC

A cartoonist, instructed by the authors, created representative schema-specific cartoons, to be presented on the same page along with the five selected items for each schema, with the aim of visually supporting the comprehensibility of each schema’s content. Thus, the preliminary illustrated questionnaire was created, consisting of 18 cartoons and 5 items each, representing the 18 schemas defined by Young (Young, 2005b). With each item phrased as a self-statement, the questionnaire utilized a four-point rating scale to assess the level of agreement (4 = “always true,” 3 = “often true,” 2 = “seldom true,” and 1 = “never true”). The questionnaire contained nine positively formulated items, whose scores had to be inverted prior to data analysis, so that high total scores for this questionnaire indicated high intensity of maladaptive schemas. To provide an economic instrument for the assessment of Young’s original 18 schemas during childhood, our aim was to reduce the number of items to the absolute minimum for successful model identification (two items per schema; Raubenheimer, 2004). Figure 1 shows a sample page including the cartoon (depicted here: defectiveness/shame) with the additional explanatory text and the two final DISC items (see below).

#### SIC

The Schema Inventory for Children (SIC) is a self-report questionnaire (retest reliability *r* = 0.67), based on Young’s 15-schema model developed by Rijkeboer and de Boo (2010). The SICs psychometric properties suggest a satisfying model fit, adequate discriminant validity, and acceptable reliability. It contains 40 items, covering 11 schemas, including 3–6 items each, that refer to children’s current noxious experiences using a 4-point-Likert scale (“not true” to “yes definitely”). With kind permission of the first author of SIC, a German language version of SIC was created and used, with the translation being counter-checked by a bi-lingual speaker of Dutch and German.

#### SDQ

The Strengths and Difficulties Questionnaire (SDQ) is a screening questionnaire to assess 3- to 16-year-old children (retest reliability *r* = 0.62). Although several variants of the SDQ are available, we used an observer-based approach (assessment by parents) for all participating children. For some children, an additional self-assessment version of the questionnaire, applicable to 11- to 16-year-old children (Goodman, 1997) was also used. Both SDQ-versions (for parents and for children) consisted of 25 items on psychological attributes, covering (1) emotional symptoms, (2) behavioral problems, (3) hyperactivity/inattention, (4) peer relationship problems, and (5) prosocial behavior. Each of the subscales is composed of five items, with subscales (1) to (4) adding up to a total difficulties score. In the present investigation, total difficulties scores from children’s self-assessment and parent versions were used to test the predictive validity of the DISC.

### Procedure

Data collection (DISC, SIC, and SDQ) took place in the classrooms of the participating schools and was scheduled to take 90 min to complete. The children were free to refuse or withdraw participation at any time, but none of them did so. For children with reading difficulties (e.g., dyslexia), extra help was provided by an accompanying research assistant (student of psychology), reading the items to them. All children received standardized instructions. To fill out the DISC, the children were asked to look at the cartoon (pictorial representation of the schema) and the contextual information provided by the protagonist’s thought bubbles or adjoining comments, then to read each item carefully and choose the answer fitting themselves the best on the 4-point rating scale. Alongside that, parents were asked to fill out the parental SDQ at home, resulting in a sample of 554, and in some classes, children were instructed to fill in the self-assessment version, resulting in a sample of 138. In order to investigate the convergent validity of the DISC, 206 children of the total answered the SIC in addition. The presentation of the above-mentioned questionnaires was largely counterbalanced to avoid sequential effects.

With the surveys being conducted in a classroom setting, all participating children of the same class took part at the same time. Pseudonym codes were used to identify the participants in case a participant’s statement indicated an emergency (e.g., feeling threatened to be murdered), enabling researchers, teachers, and caregivers to identify the child and offer immediate help. However, no such emergency occurred. Having completed the questionnaires, all children present in the classroom received a piece of candy, playful pencil, or a bouncy ball.

### Statistical analyses

#### DISC model validation

The dimensionality of the DISC was evaluated using confirmatory factor analysis (CFA) of the preliminary 90-item version, executed with SPSS Amos (IBM Corp., 2015). Designing a short instrument applicable to young schoolchildren, the aim was to select two items per schema only, the minimum for successful model identification (Raubenheimer, 2004). Therefore, CFAs were first conducted on a factor level to identify items with high factor loadings and low standardized residuals. Distributional characteristics (right skewness) and item difficulty (*p*_m_ < 0.5) were also taken into account, considering the clinical implications of Young’s EMS theory. Thus, 36 observed variables were to be selected and used to model the latent variables of Young’s original 18 factors. Additionally, as maladaptation is supposed to be a common denominator for all of Young’s proposed early maladaptive schemas, it was assumed that the 18 factors would converge into a general factor. All the participant’s data were included for data analyses.

#### Test-retest reliability

For the evaluation of the DISC’s test-retest reliability, 76 children were asked to participate in a retest and assigned to one of two interval groups: Group 1 with an interval length of 13 to 14 months (*n* = 36), and Group 2 with an interval length of 26 to 34 months (*n* = 36). To analyze the test-retest reliability Pearson’s correlation coefficients were calculated for each interval. Calculations were carried out utilizing the Statistical Package of the Social Sciences (IBM Corp., 2015).

#### Criterion validity

To investigate the convergent validity, the results of the DISC and the SIC were compared in 206 children. For the eight original EMS factors, measured by both the DISC and the SIC, Pearson’s correlations were used to check for convergent validity. Regarding the three combined factors included in the SIC, a composition of the analogue DISC factors was used.

To investigate predictive validity and estimate the DISC’s relation to children’s behavioral problems, correlations between DISC scores and results of the SDQ were calculated. This was done for the 554 parent versions (other-reported) and 152 children versions (self-reported) of the SDQ.

## Results

### DISC model validation

After inspection of item characteristics and analyses of the hypothesized latent 18-factor structure of the 90-item preliminary version of the DISC, a 36-item short form was compiled, consisting of two items per schema using the criteria for item selection (factor loadings, standardized residuals, item distribution, and item difficulty). The resulting model was tested for an acceptable fit (CFA, maximum likelihood). Root mean square error of approximation (RMSEA; ~ cut-off < .06), standardized root mean square residual (SRMR; ~ cut-off < .05), and comparative fit index (CFI; ~ cut-off > .90) were utilized as subjective indices of goodness-of-fit. Furthermore, the Tucker-Lewis-Index (TLI; cut-off > .90) was calculated, although it tends to penalize complex models. Regarding the *χ*^2^ statistic of the CFA, it is advised to evaluate *χ*^2^ for larger samples in comparison with the degrees of freedom (~ cut-off *χ*^2^/df < 2) (Mueller, 1996).

Thus, all 18 original factors proposed by Young (2005a) were represented in the resulting model and tested for goodness-of-fit. Furthermore, all 18 factors were assumed to converge into a general factor representing maladaptation. Some of the evaluated indices pointed to a good to acceptable fit (RMSEA = 0.04; SRMR = 0.05), while others just missed out on fulfilling the set criteria (*χ*^2^/df = 2.05; CFI = 0.86, TLI = .85). Nonetheless, considering the structural complexity, the results indicate a sufficient basis for the 18-factor model. A summary of the fit indices can be found in Table 2.

**Table 2:** Results of the confirmatory factor analyses for the DISC: 18 schema-based factors plus superordinate general factor

	Fit indices						
	*χ* ^*2*^	df	*χ*^*2*^*/*df	*RMSEA* (90% CI)	*CFI*	*TLI*	*SRMR*
*N* = 569	1178.48	576	2.05	.04 (.039–.046)	.86	.85	.05

Table 3 provides an overview of the parameters within the model on both the schema and item levels. Overall, the results highlight a moderate to good factorial validity, with *p* values indicating a level of significance smaller than .001 for each path coefficient. For the general factor, the standardized regression weights of the subscales ranged from .43 (abandonment) to .98 (negativity/pessimism; *M* = .62, *SE* = .03). On schema-item level, *λ* ranged from .28 to .90 (*M* = .64, *SE* = .02). However, with the explained variance *R*^2^ ranging from .19 (abandonment) to .96 (negativity/pessimism), the maladaptive significance of the EMS appear to vary greatly (*M* = 34.20, *SE* = 4.20). On the schema-item level, this variance is even more apparent (range .08 to .81; *M* = .43, *SE* = .03). The mean squared multiple correlation is greater than .40 and thus indicates that the variables share a substantial amount of variance with the underlying theoretical construct (Taylor & Todd, 1995), but only 12 of the 36 items show *R*^2^ values greater than .50. Although this might be related to the developmental aspects of EMS (or to the limited number of items, for that matter), the convergent validity of some items must be considered questionable.

**Table 3:** Parameters within the model: descriptive statistics, standardized regression weights (*λ*), squared multiple correlations (*R*^2^) from the CFA on schema and item level

Schema		G-factor level		Items			Schema level	
	*M*_*SUM*_ (*SE*)	Λ	*R* ^2^		*M (SE)*	Range	λ	*R* ^2^
Insufficient self-control	3.96 (0.06)	.50 ***	.25	Item 01	1.87 (0.04)	1–4	.73 ***	.54
				Item 02	2.09 (0.04)	1–4	.44 ***	.20
Subjugation	3.90 (0.06)	.52 ***	.28	Item 03	2.02 (0.04)	1–4	.55 ***	.30
				Item 04	1.88 (0.03)	1–4	.47 ***	.22
Mistrust/abuse	3.38 (0.06)	.73 ***	.53	Item 05	1.69 (0.03)	1–4	.78 ***	.60
				Item 06	1.68 (0.03)	1–4	.70 ***	.49
Defectiveness/shame	2.80 (0.05)	.73 ***	.53	Item 07	1.44 (0.03)	1–4	.60 ***	.37
				Item 08	1.36 (0.03)	1–4	.78 ***	.61
Social isolation/alienation	2.98 (0.05)	.57 ***	.33	Item 09	1.50 (0.04)	1–4	.49 ***	.24
				Item 10	1.47 (0.03)	1–4	.66 ***	.43
Dependence/incompetence	3.71 (0.06)	.59 ***	.34	Item 11	1.81 (0.03)	1–4	.72 ***	.53
				Item 12	1.90 (0.04)	1–4	.62 ***	.38
Failure	3.40 (0.06)	.64 ***	.41	Item 13	1.95 (0.04)	1–4	.70 ***	.50
				Item 14	1.46 (0.03)	1–4	.76 ***	.58
Vulnerability	3.74 (0.06)	.60 ***	.37	Item 15	1.73 (0.03)	1–4	.83 ***	.68
				Item 16	2.01 (0.04)	1–4	.71 ***	.50
Enmeshment	4.60 (0.06)	.50 ***	.25	Item 17	2.64 (0.04)	1–4	.66 ***	.43
				Item 18	1.96 (0.04)	1–4	.65 ***	.42
Entitlement/grandiosity	2.81 (0.04)	.68 ***	.46	Item 19	1.58 (0.03)	1–4	.52 ***	.27
				Item 20	1.24 (0.02)	1–4	.28 ***	.08
Emotional deprivation	2.96 (0.06)	.54 ***	.29	Item 21	1.40 (0.03)	1–4	.55 ***	.30
				Item 22	1.57 (0.03)	1–4	.90 ***	.81
Abandonment/instability	3.07 (0.05)	.43 ***	.19	Item 23	1.45 (0.03)	1–4	.60 ***	.30
				Item 24	1.62 (0.03)	1–4	.80 ***	.64
Self-sacrifice	3.91 (0.06)	.75 ***	.57	Item 25	2.33 (0.04)	1–4	.59 ***	.34
				Item 26	1.59 (0.03)	1–4	.67 ***	.45
Approval-seeking	3.18 (0.05)	.48 ***	.23	Item 27	1.43 (0.03)	1–4	.71 ***	.51
				Item 28	1.75 (0.04)	1–4	.59 ***	.35
Negativity/pessimism	3.19 (0.05)	.98 ***	.96	Item 29	1.60 (0.03)	1–4	.67 ***	.45
				Item 30	1.58 (0.03)	1–4	.63 ***	.39
Emotional inhibition	4.14 (0.06)	.63 ***	.40	Item 31	1.97 (0.04)	1–4	.64 ***	.40
				Item 32	2.17 (0.04)	1–4	.64 ***	.41
Punitiveness	3.37 (0.06)	.63 ***	.40	Item 33	1.77 (0.04)	1–4	.79 ***	.63
				Item 34	1.60 (0.03)	1–4	.52 ***	.27
Unrelenting standards	4.05 (0.06)	.57 ***	.33	Item 35	1.82 (0.04)	1–4	.70 ***	.49
				Item 36	2.24 (0.04)	1–4	.45 ***	.20

Note: *p < .05, **p < .01, ***p < .001

Regarding its sum score, the internal consistency of the DISC appears to be good, with a Cronbach’s alpha of .87, and there was no increase in Cronbach’s alpha when any item was excluded. The correlations between the schema-based factors and the superordinate general factor are shown in Table 4. While all 18 schema-based factors are significantly related to the general factor (each with *p* < .001), the intercorrelations show a number of distinct exceptions (*r* < .05). As the overall sum score shows significant correlations with each of the 18 schema-based factors and, additionally, no negative correlations were found, its interpretation as an indicator of dysfunctionality, covering distinctive aspects of maladaptation, seems plausible.

**Table 4:** Intercorrelations between the DISC’s overall sum score and the sum scores on schema level (*N* = 569). According significance levels (*p* values) are indicated in the lower triangular part of the matrix

	0	1	2	3	4	5	6	7	8	9	10	11	12	13	14	15	16	17	18
0. DISC sum score		.44	.40	.62	.60	.43	.54	.56	.55	.46	.41	.51	.39	.59	.44	.72	.53	.56	.49
1. Insufficient self-control (IS)	***		.15	.22	.19	.14	.30	.22	.18	.11	.14	.17	.16	.15	.17	.26	.20	.19	.10
2. Subjugation (SB)	***	***		.18	.21	.12	.13	.22	.13	.19	.13	.14	.11	.31	.04	.26	.13	.19	.07
3. Mistrust/abuse (MA)	***	***	***		.38	.26	.27	.32	.33	.20	.26	.27	.25	.42	.22	.45	.27	.26	.24
4. Defectiveness/shame (DS)	***	***	***	***		.25	.27	.38	.24	.22	.19	.35	.30	.30	.23	.43	.28	.22	.16
5. Social isolation/alienation (SI)	***	***	**	***	***		.13	.16	.22	.07	.24	.21	.21	.23	.02	.29	.30	.16	.10
6. Dependence/incompetence (DI)	***	***	***	***	***	**		.30	.32	.27	.14	.19	.10	.19	.23	.32	.18	.31	.27
7. Failure (FA)	***	***	***	***	***	***	***		.27	.17	.08	.25	.18	.25	.17	.44	.26	.28	.18
8. Vulnerability (VU)	***	***	***	***	***	***	***	***		.28	.08	.20	.13	.27	.12	.45	.22	.26	.25
9. Enmeshment (EM)	***	**	***	***	***	.11	***	***	***		.21	.08	.01	.32	.16	.27	.12	.27	.20
10. Entitlement/grandiosity (ET)	***	***	**	***	***	***	***	.08	.06	***		.19	.19	.26	.20	.28	.16	.15	.19
11. Emotional deprivation (ED)	***	***	***	***	***	***	***	***	***	.05	***		.35	.25	.19	.31	.17	.20	.22
12. Abandonment/instability (AB)	***	***	**	***	***	***	*	***	**	.87	***	***		.11	.03	.26	.20	.10	.02
13. Self-sacrifice (SS)	***	***	***	***	***	***	***	***	***	***	***	***	**		.21	.41	.25	.32	.25
14. Approval-seeking (AS)	***	***	.38	***	***	.61	***	***	**	***	***	***	.45	***		.25	.19	.27	.35
15. Negativity/pessimism (NP)	***	***	***	***	***	***	***	***	***	***	***	***	***	***	***		.41	.36	.27
16. Emotional inhibition (EI)	***	***	**	***	***	***	***	***	***	**	***	***	***	***	***	***		.26	.22
17. Punitiveness (PU)	***	***	***	***	***	***	***	***	***	***	***	***	*	***	***	***	***		.29
18. Unrelenting standards (US)	***	*	.12	***	***	*	***	***	***	***	***	***	.71	***	***	***	***	***	

Note: *p < .05; **p < .01; ***p < .001

### Reliability

For the test-retest interval of 13 to 14 months, Pearson’s correlations of the DISC sum scores revealed a highly significant positive correlation (*r* (36) = .61, *p* < .001). For the test-retest interval of 26 to 34 months, results still showed a significant correlation, albeit at a lower level of significance (*r* (36) = .37, *p* < .05).

### Criterion validity

To evaluate criterion validity, outcomes for the eight original schemas included in both the DISC and SIC were taken into consideration. Highly significant correlations between the schema-associated scores of the DISC and the SIC support the validity of the DISC structure (Table 5). Comparable results were found for the correlations between the three additional SIC factors *Loneliness*, *Vulnerability*, and *Submission* and their composite counterparts based on the corresponding DISC schemas.

**Table 5:** Criterion validity: correlations between the factors proposed by the SIC and their DISC counterparts (*N* = 206)

SIC factors	DISC sum score correlation	
	*r* (206)	*p*
Insufficient self-control (IS)	.29	***
Mistrust/abuse (MA)	.59	***
Defectiveness/shame (DS)	.46	***
Failure (FA)	.69	***
Enmeshment (EM)	.29	***
Entitlement/grandiosity (ET)	.22	***
Self-sacrifice (SS)	.28	***
Unrelenting standards (US)	.35	***
Loneliness (comprised ED, SI)	.52	***
Vulnerability (comprised AB, VU)	.57	***
Submission (comprised SB, EI, DI)	.52	***

Note: *p < .05, **p < .01, ***p < .001

The SIC measures eight of the factors proposed by Young plus three factors, build on two to three of the original schemas

Correlations between the DISC sum score and the SDQ’s total difficulties scores (parent and self-assessment version) confirm the predictive validity of the DISC, but the correlation was higher for the self-assessment version (*r* (152) = .48, *p* < .001) than for the parent version of the SDQ (*r* (552) = .23, *p* < .001). Interestingly, the correlation between both SDQ scores misses statistical significance, if only by a small margin (*r* (136) = .17, *p* = .053).

## Discussion

The present study served to describe the development and evaluation of DISC, an illustrated questionnaire to assess maladaptive schemas in children and its reliability, validity, and dimensionality. The questionnaire displayed a highly significant test-retest reliability over a period of 13 to 14 months, and a lower but nevertheless significant test-retest reliability over a period of 26 to 34 months. Ratings on the schemas of the DISC were highly significantly correlated with ratings on the comparable schemas of the SIC, a comparable and evaluated questionnaire, thus confirming the construct validity of the DISC. In addition, ratings on the DISC were significantly correlated with scores on the SDQ, thus indicating the predictive validity of the DISC for behavioral problems. Regarding the dimensionality of the DISC, its latent structure yielded the hypothesized 18-factors, with two items per schema. Thus, all 18 original schemas proposed by Young (2005b) were represented in the resulting model and tested for goodness-of-fit. Furthermore, it could be shown, that all 18 factors converged to one general factor representing maladaptation. The results show that even children of around 8 to 13 years of age exhibit the same schemas as were identified for adults.

At present, several studies support the 18-factor structure, in factor analyses of schema questionnaires in adults (Calvete, Orue, & Gonzalez-Diez, 2013; Hawke & Provencher, 2012; Kriston et al., 2013; Lee et al., 2015; Saariaho et al., 2009). In contrast to most other schema questionnaires for children and adolescents, in which only 15 schemas were investigated, we also confirmed 18 schemas for children. This would support Young et al.’s (2003) notion, that EMS are already developed in (early) childhood. Since former studies (Beckley, 2002; Schmidt et al., 1995; Stallard & Rayner, 2005; Van Vlierberghe et al., 2010; Welburn et al., 2002) were also able to find and confirm to a large extent all of the schemas they had investigated, it can be assumed that they would have been able to detect more schemas if only they would have challenged this issue.

Stallard (2007) and Rijkeboer and de Boo (2010) found that not all of the schemas in their questionnaires were predictive for psychopathology and assumed that some schemas in childhood might not be maladaptive, at least not at this stage of life. This finding leads to the question, whether the 18 schemas of Young, which are reflected in the 18 schemas of the DISC, not only indicate maladaptive but also adaptive schemas (neutral or even positive schemas). Arguments against the latter consideration are that we found a high internal consistency for the DISC (Cronbach’s alpha of .87) and a general factor in the factor analysis, indicating that the assessed schemas converge in one direction. All 36 items loaded high on Cronbach’s alpha, and there was no increase in Cronbach’s alpha when any item was excluded, indicating that the items have at least one important factor in common. We would call this general factor “dysfunctionality” or “maladaptation”.

On the question of why we were able to identify all 18 schemas (by using a CFA) with the DISC, whereas the SIC’s analysis yielded only eight original and three composed schemas? Bearing in mind that the three composed schemas are mixtures of the remaining seven schemas, which are left from the 15 investigated ones, we might want to take into account that in the SIC analysis the sample was divided into two groups, in order to use a principal factor analysis, and afterwards a CFA. We decided to do only the CFA with the full sample (*N* = 569) because of the high convergence that was yielded by the DISC’s and SIC’s schemas and further the comparable sociodemographic variables in both studies (age, gender). In that sense, we would like to propose an interpretation of the DISC study as a kind of extension of the SIC, giving way to a method where the whole sample is used for CFA, with the results therefore having much greater power to detect factors that we would not have been able to find if analysis had only included half of the sample.

Probably, the most important difference between the DISC and the SIC and other questionnaires like the SQC (Stallard & Rayner, 2005) and SQS (Güner, 2016) is that we put a large emphasis on the visual illustration of our schema items, using cartoons, which were especially created for the sake of the schema illustration and, specifically, for this study. In our opinion, and also due to the children’s positive feedback, these pictures were highly attractive and gave them a much better understanding of what was meant when reading the items.

The test-retest reliability is high compared to the reported 6-month retest reliability of Stallard (2007), whose coefficients were only modest (range *r* .27–.54). However, our results are in line with the SIC’s reliability of .67 (though the interval of 4 weeks is much smaller). The relatively high retest-reliability of the DISC might be due to the cartoons that explained each schema in a visual way. However, with regards to the participants’ young age, aspects of natural development should be considered in the evaluation of the test-retest reliability, as changes over the course of months or years could be due to experiences and life events, not only limitations in the reliability of the instrument.

Concerning the convergent validity of the DISC, we found that the correlation with the self-assessment version of the SDQ was much higher (*r* = .48) than that for the parent version of the SDQ (*r* = .23), indicating that children are closer to their own thoughts, emotions, and finally behavior than their parents are. Interestingly, the correlation between the parent’s and the child’s total difficulty score reveals a low correlation coefficient of *r* = .17, missing even a statistical significance. We interpret this result with the notion that children’s behavior observed from outside is not necessarily to be connected with the children’s own perception. We conclude that it is worthwhile to accept the importance of taking into account children’s answers as an important source when investigating how children feel and think about themselves, in order to understand the resulting behavior.

To address some limitations of the study, we examined only a community sample of school children, though assessment of schemas originally focused on identifying persons at risk of psychopathology. As we know from prevalence studies about psychic disorders in childhood and adolescence, 15–20% have already developed or are at least at risk of developing a disorder (Barkmann & Schulte-Markwort, 2012). Thus, the DISC should also be tested in a clinical sample of children in the future.

Regarding the school’s low response rate of about 13% suggest a lack of representativeness. Asked for the reason of refusal, all denying school’s directors argued that their schools would already participate in other studies and/or are overloaded. Though the low response rate seems to be a strong limiting factor of the study, the highly correlated scores of the DISC with the well-evaluated SIC suggest a comparable sample of these two studies (SIC and DISC).

For future studies, we would like to propose to offer schools high incentives (e.g., of financial nature) or to include the study into another study, to limit a possible non response bias on the data.

Another improvement of the study would be to evaluate the cartoons separately. Although the children’s (informal) feedback concerning the “visual explanation” of the DISC items was extremely positive, it is not clear what effect they really had on the comprehensiveness of the item’s content and willingness to answer as honestly as possible.

In summation, the present study on an illustrated schema questionnaire for children showed that the 18 EMS as described by Young can also be observed in children. However, it seems reasonable that these schemas are not independent of each other since they converge into a general factor, as discovered by CFA. The newly developed DISC seems thus to be a reliable and valid instrument to assess maladaptive schemas in children. Nonetheless, we suggest being cautious with the interpretation of the DISC schemas because of the low item number of two per schema and because of the partly low correlation scores. Although it can be assumed that in children, the EMS are not as stable as in adults since they are developing in these years and probably not fully expressed, it seems useful to investigate these schemas and their course of development during life or under challenging circumstances. The inclusion of a clinical sample might also enlarge the range of data, leading to a more specific knowledge about specific disorders (e.g., ADHD) and typical schemas. The DISC may thus be an instrument to obtain information about these developmental and disorder specific aspects.

## Conclusions

The paper describes the development and evaluation of an illustrated questionnaire to assess schemas in children. It is the first Schema Questionnaire that is illustrated with specific schema-related cartoons. All 18 early maladaptive schemas (EMS) according to Young are confirmed by a CFA, indicating that even children of around 8 to 13 years of age exhibit the same schemas as were identified for adults. Ratings on the DISC’s schemas were highly significantly correlated with ratings on the comparable schemas of the SIC (Rijkeboer & de Boo, 2010), which is a comparable and well-evaluated questionnaire. In addition, ratings on the DISC were significantly correlated with scores of the Strengths and Difficulties Questionnaire (SDQ; Goodman, 1997), indicating predictive validity of the DISC for behavioral problems. Since the questionnaire is a short an economic instrument to assess schemas in children, and the results reveals high test-retest reliability as well as confirmed construct and predictive validity, it might be a promising tool to assess schema dispositions already early in childhood, when they are going to develop. That would allow an adequate treatment in time, before EMS become stable in adulthood, and show its devastating effects on mental health.

## Appendix

Thirty-six items of DISC (in parenthesis the abbreviation of the respective schemas they present; see Tables 1 or 4):

1. I get upset really quickly if something takes longer than intended. (IS)
2. I cannot stand waiting for something. (IS)
3. I prefer letting other people decide, because I do not want get into conflict. (SB)
4. The opinion of others is more important to me than my own. (SB)
5. I think that my friends will betray me sooner or later. (MA)
6. I think that other people take advantage of me. (MA)
7. I cannot understand how someone can like me. (DS)
8. I am not worth to be loved. (DS)
9. I do not like spending time with other people. (SI)
10. I prefer to stay on my own, rather than joining a group. (SI)
11. I need a lot of support in my daily routine. Otherwise, I become overtaxed. (DI)
12. Without the help of my parents I can hardly do anything. (DI)
13. No matter what I do at school, others are always better than me. (FA)
14. My performance is poor, and it will always remain so. (FA)
15. I have the feeling that any moment could turn into a catastrophe. (VU)
16. I am afraid that something bad might happen. (VU)
17. When my parents have problems, I instantly feel bad. (EU)
18. I feel responsible for the lives of my parents. (EU)
19. Others call me the know-it-all. (ET)
20. Others should do what I want. (ET)
21. I don’t get any attention or love. (ED)
22. No one really takes time for me. (ED)
23. I’m sure that my family and friends will always be there for me. (Inverted item, AB)
24. I believe that my family and friends will stay by my side in every situation. (Inverted item, AB)
25. If you ask me for help, I’ll do anything, even if I’m incapable of doing it. (SS)
26. I have no time for myself, because I take care of others all the time. (SS)
27. It’s important for me that people around me tell how great I am. Otherwise I don’t feel good. (AS)
28. Owning modern clothes and knowing cool people, gives me the feeling of being special. (AS)
29. Most of the things in my life are bad or will turn out badly. (NP)
30. I’m not good at taking decisions, because I’m scared of the consequences. (NP)
31. Showing feelings is totally embarrassing. (EI)
32. Others are not supposed to know when I’m anxious, angry or sad. (EI)
33. If I make mistakes, I deserve to be punished. (PU)
34. There must be some kind of punishment! This applies to all those who make mistakes, it does not matter whether it is done intentionally or unintentionally. (PU)
35. I put myself under a lot of pressure to show me and the others, how good I am. (US)
36. The most important thing in my life is to be good at school. (US)

## Authors’ information

No further information.
